# Fully automated analysis combining [^18^F]-FET-PET and multiparametric MRI including DSC perfusion and APTw imaging: a promising tool for objective evaluation of glioma progression

**DOI:** 10.1007/s00259-021-05427-8

**Published:** 2021-06-25

**Authors:** K. J. Paprottka, S. Kleiner, C. Preibisch, F. Kofler, F. Schmidt-Graf, C. Delbridge, D. Bernhardt, S. E. Combs, J. Gempt, B. Meyer, C. Zimmer, B. H. Menze, I. Yakushev, J. S. Kirschke, B. Wiestler

**Affiliations:** 1 Department of Neuroradiology, TUM School of Medicine, Klinikum Rechts Der Isar, Technical University of Munich, Ismaninger Str. 22, 81675 Munich, Germany; 2 Department of Nuclear Medicine, TUM School of Medicine, Klinikum Rechts Der Isar, Technical University of Munich, Ismaninger Str. 22, 81675 Munich, Germany; 3 Department of Neurology, TUM School of Medicine, Klinikum Rechts Der Isar, Technical University of Munich, Ismaninger Str. 22, 81675 Munich, Germany; 4 Department of Neuropathology and Pathology, TUM School of Medicine, Technical University of Munich, Trogerstr.18, 81675 Munich, Germany; 5 Department of Radiation Oncology, TUM School of Medicine, Klinikum Rechts Der Isar, Technical University of Munich, Ismaninger Str. 22, 81675 Munich, Germany; 6 Department of Radiation Sciences (DRS), Institute of Radiation Medicine (IRM), Ingolstädter Landstraße 1, Neuherberg, Germany; 7 Deutsches Konsortium Für Translationale Krebsforschung (DKTK), Partner Site Munich, Munich, Germany; 8 Department of Neurosurgery, TUM School of Medicine, Klinikum Rechts Der Isar, Technical University of Munich, Ismaninger Str. 22, 81675 Munich, Germany; 9 Image-Based Biomedical Modeling, Department of Informatics, Technical University of Munich, Munich, Germany; 10 TranslaTUM (Zentralinstitut Für Translationale Krebsforschung der Technischen Universität München), Einsteinstr. 25, 81675 Munich, Germany

**Keywords:** Glioma progression, Fully automated, [^18^F]-FET-PET, Multiparametric MRI, DSC perfusion, APTw, Kirschke, J. S. and Wiestler, B. coshared last

## Abstract

**Purpose:**

To evaluate diagnostic accuracy of fully automated analysis of multimodal imaging data using [^18^F]-FET-PET and MRI (including amide proton transfer-weighted (APTw) imaging and dynamic-susceptibility-contrast (DSC) perfusion) in differentiation of tumor progression from treatment-related changes in patients with glioma.

**Material and methods:**

At suspected tumor progression, MRI and [^18^F]-FET-PET data as part of a retrospective analysis of an observational cohort of 66 patients/74 scans (51 glioblastoma and 23 lower-grade-glioma, 8 patients included at two different time points) were automatically segmented into necrosis, FLAIR-hyperintense, and contrast-enhancing areas using an ensemble of deep learning algorithms. In parallel, previous MR exam was processed in a similar way to subtract preexisting tumor areas and focus on progressive tumor only. Within these progressive areas, intensity statistics were automatically extracted from [^18^F]-FET-PET, APTw, and DSC-derived cerebral-blood-volume (CBV) maps and used to train a Random Forest classifier with threefold cross-validation. To evaluate contribution of the imaging modalities to the classifier’s performance, impurity-based importance measures were collected. Classifier performance was compared with radiology reports and interdisciplinary tumor board assessments.

**Results:**

In 57/74 cases (77%), tumor progression was confirmed histopathologically (39 cases) or via follow-up imaging (18 cases), while remaining 17 cases were diagnosed as treatment-related changes. The classification accuracy of the Random Forest classifier was 0.86, 95% CI 0.77–0.93 (sensitivity 0.91, 95% CI 0.81–0.97; specificity 0.71, 95% CI 0.44–0.9), significantly above the no-information rate of 0.77 (p = 0.03), and higher compared to an accuracy of 0.82 for MRI (95% CI 0.72–0.9), 0.81 for [^18^F]-FET-PET (95% CI 0.7–0.89), and 0.81 for expert consensus (95% CI 0.7–0.89), although these differences were not statistically significant (p > 0.1 for all comparisons, McNemar test). [^18^F]-FET-PET hot-spot volume was single-most important variable, with relevant contribution from all imaging modalities.

**Conclusion:**

Automated, joint image analysis of [^18^F]-FET-PET and advanced MR imaging techniques APTw and DSC perfusion is a promising tool for objective response assessment in gliomas.

**Supplementary Information:**

The online version contains supplementary material available at 10.1007/s00259-021-05427-8.

## Introduction

Managing glioma patients, radiologists and clinicians often need to distinguish progressive disease (PD) from treatment-related changes (TRCs). Radiologic assessment of tumor response and progression is traditionally based on volumetric changes of the enhancing tumor area. The discrimination between PD and TRC (such as blood–brain-barrier breakdown following radiotherapy) is however extremely challenging as both typically present with new or progressive contrast enhancement (CE) on T1-weighted gadolinium-enhanced magnetic resonance imaging (MRI) [1–3]. While for PD, increased contrast enhancement is typically the result of neoangiogenesis, changes in contrast enhancement after therapy can result from a variety of nontumorous processes, such as ischemia, postsurgical changes, treatment-related inflammation, subacute radiation effects, and radiation necrosis [4].

Diagnosis and treatment of PD and TRC requires multidisciplinary structures of care, and defined processes. Diagnosis has to be made on an interdisciplinary level with the joint knowledge of a neuroradiologist, radiation oncologist, neurosurgeon, and neurooncologist. A multi-step approach as an opportunity to review as many characteristics as possible to improve diagnostic confidence is recommended. Additional information about radiotherapy (RT) techniques are crucial for diagnosis. Yet, pathologic confirmation is considered the most reliable method to differentiate PD from TRC. However, to avoid unnecessary surgery, many efforts have been undertaken to improve non-invasive tumor response assessment. In view of the inability of traditional MRI including T1-weighted or T2-weighted sequences to reliably differentiate PD and TRC [5], advanced imaging modalities aiming to visualize tumor biology and key oncogenic processes have been frequently studied [6].

Relative cerebral blood volume (rCBV) obtained from MR-based dynamic susceptibility contrast (DSC) perfusion imaging has been histopathologically confirmed to provide evidence of neoangiogenesis [7] — a hallmark of malignant gliomas. Some studies have reported that median rCBV and histogram analysis of rCBV can help to differentiate TRC from PD [8, 9] by distinguishing between vital tumor tissue in PD and other causes of contrast enhancement in TRC.

A relatively novel but promising molecular MRI technique is amide proton transfer-weighted (APTw) imaging. This imaging method capitalizes on the constant dissociation and transfer of amide-bound hydrogen atoms into the surrounding water. Previous studies proofed the benefit of APTw imaging in the differentiation between the different WHO grades of gliomas [10] as well as the ability to differentiate between tumor progression and therapy-related changes [11, 12].

With FET as a tracer, PET is known to visualize the amino acid uptake in gliomas and thus metabolically active tumor cells [13]. Several studies exist that demonstrated the clinical utility of [^18^F]-FET-PET for preoperative grading [14] and biopsy planning [15] as well as for differentiating tumor progression from treatment-related changes [16].

Combining information from different techniques and imaging modalities can help to further decipher the complex diagnosis of progress and TRC in gliomas. A few studies have already investigated the added value of APTw to diffusion- and perfusion-weighted imaging [17] or methionine PET [18]. However, traditional assessment of multimodal imaging data is challenging: Extracting (semi-)quantitative information (such as tumor-to-background ratio (TBR)) from such images is observer-dependent despite means to better standardize this. Further, truly integrating imaging information from multiple modalities (in a region- or even voxel-wise fashion) requires high proficiency in reading these images [19].

In view of recent advances in deep learning for medical image analysis, we developed a fully automated pipeline for longitudinal assessment of changes in tumor morphology, unbiased extraction of quantitative imaging information in these areas from multimodal, advanced imaging modalities, and ultimately predictive modeling of response assessment. Here, we evaluated the accuracy of such a model-based differentiation between PD and TRC [20].

## Material and methods

### Patient data

All patients were part of a consecutive, prospective observational glioma cohort from December 2017 to April 2020, approved by our local Institutional Review Board. All patients gave written informed consent.

**Fig. 1:**
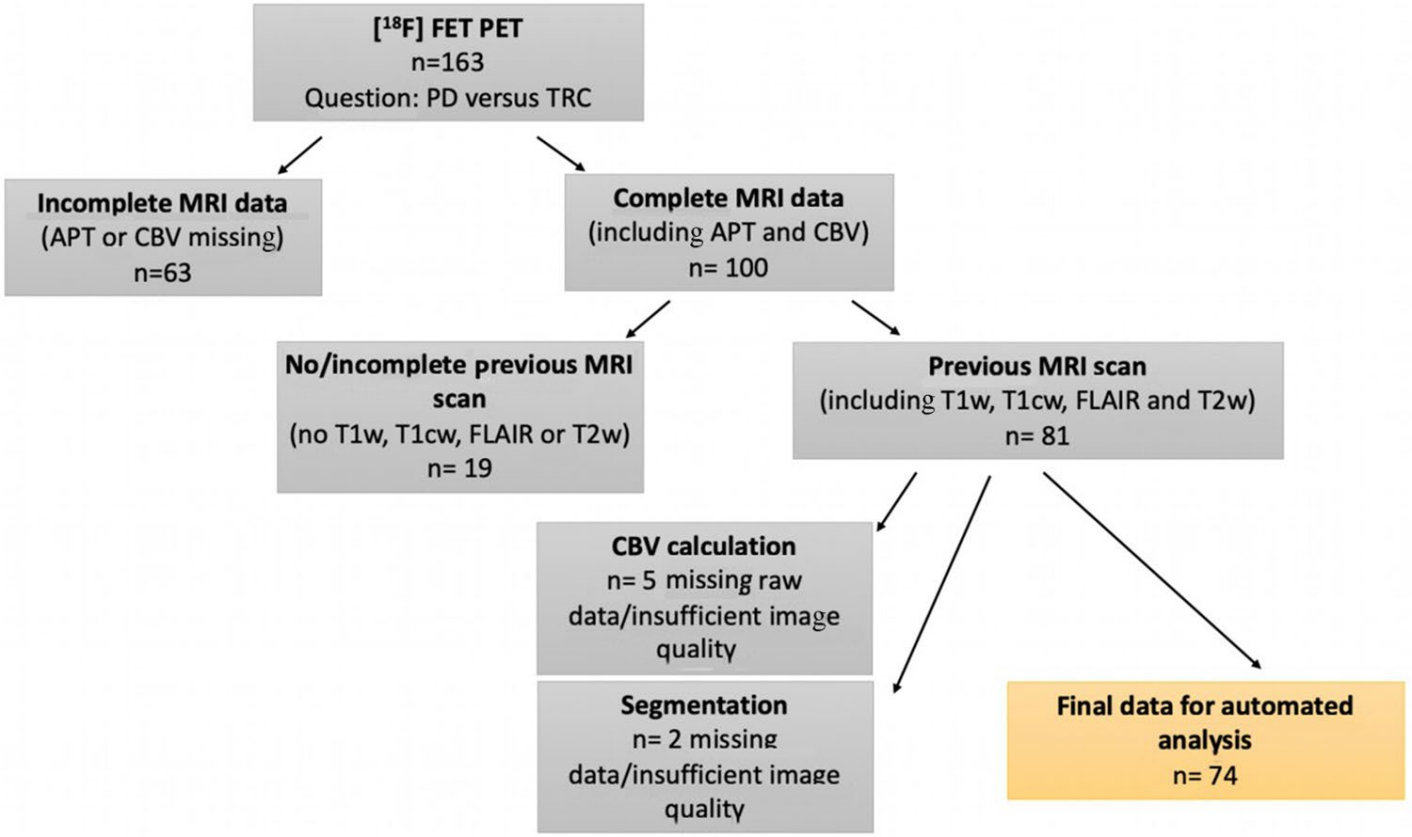
Flow chart patient selection: From initial 163 [^18^F]-FET-PET data sets performed to differentiate PD vs. TRC, we excluded 89 data sets due to missing data or insufficient image quality

For this retrospective study, we analyzed data of 66 patients (74 MRI and [^18^F]-FET-PET scans) with histologically confirmed glioma (WHO grades I–IV) according to the 2016 WHO classification of CNS tumors [21]. We included all patients in this time frame who (a) had available MR imaging (including T2- and T1-weighted imaging before and after administration of gadolinium-based contrast agent plus APTw imaging and DSC perfusion) and who (b) had suspected tumor progression with consecutively performed [^18^F]-FET-PET, (c) with interdisciplinary consensus reading in our neurooncology tumor board, and (d) where either histopathological or additional long-term follow-up confirmation of the diagnosis (PD or TRC) was available (Fig. 1). All images were rated according to the RANO criteria [22], and cases with a morphologically mixed appearance in MRI and/or PET scan (containing both areas with PD and TRC) were accordingly rated as progressive disease (MRI 4.1%; 3/74 and [^18^F]-FET-PET 8.1%; 6/74). MRI and PET assessment were taken from the clinical report. Eight patients were included twice and analyzed as separate cases as they fulfilled inclusion criteria at two different points of time (between the two included scans, four of them underwent resection, three chemotherapy, and one radio-chemotherapy). Further patient characteristics are given in Table 1.

**Table 1:** Patient and tumor characteristics

Sex (m/w)	55%; 45%; (41; 33)
Age (mean ± SD in years)	54.91 ± 12.2
Diagnosis	
Glioblastoma WHO IV	69%; 51/74
Astrocytoma WHO II	3%; 2/74
Astrocytoma WHO III	9%; 7/74
Oligodendroglioma WHO II	3%; 2/74
Oligodendroglioma WHO III	15%; 11/74
Pilocytic astrocytoma WHO I	1%; 1/74
Molecular parameters	
IDH	
Wildtype	64%; 47/74
Mutant	33%; 25/74
N/A	3%; 2/74
1p/19q	
Co-deleted	15%; 11/74
Intact	11%; 8/74
N/A	74%; 55/74
MGMT	
Methylated	51%; 38/74
Unmethylated	39%; 29/74
N/A	10%; 7/74
Prior therapy	
Surgery	100%, 74/74
Radiotherapy	95%; 70/74
Subsequent surgery	58%; 43/74
Mean interval (PET to subsequent surgery)	12 ± 10 days
Mean interval to previous MRI	102 days
Mean interval from prior RT to MRI	544 days
Mean interval from prior RT to PET	559 days

### Image acquisition

[^18^F]-FET-PET data were acquired on a PET/MR (Biograph mMR, Siemens Healthcare GmbH, Erlangen, Germany), n = 57, and a PET/CT (Biograph mCT; Siemens Healthcare, Knoxville, TN, USA), n = 24, according to a standard clinical protocol. Patients were asked to fast for a minimum of 4 h before scanning. Emission scans were acquired at 30 to 40 min after intravenous injection of a target dose of 185 ± 10% MBq [^18^F]-FET. Attenuation correction was performed according to vendor’s protocol.

The MR imaging was performed on a Philips (Best, the Netherlands) 3 T scanner (Achieva or Ingenia). Our MR protocol included an isotropic FLAIR (voxel size 1 mm^3^, TE = 269 ms, TR = 4800 ms, TI = 1650 ms), isotropic T1-TFE (voxel size 1 mm^3^, TE = 4 ms, TR = 9 ms) before and after contrast, axial T2 (voxel size 0.36 × 0.36 × 4 mm^3^, TE = 87 ms, TR = 3396 ms), 3D APTw (fast spin echo, voxel size 0.9 × 0.9 × 1.8 mm^3^, TE = 7.8 ms, TR = 6 s, RF saturation pulse train B_1,rms_ = 2 μT, T_sat_ = 2 s, duty-cycle 100%, 9 volumes ω =  ± 3.5 ppm ± 0.8 ppm and reference ω_0_ =  − 1560 ppm, intrinsic B_0_ correction [23], MTR asymmetry at + 3.5 ppm as APT-weighted = APTw contrast), as well as DSC perfusion (voxel size 1.75 × 1.75 × 4 mm^3^, TE = 40 ms, TR = 1547 ms, Flip Angle = 75°, 80 dynamics).

### Image processing

Processing of DSC data for rCBV parameter maps used custom programs in MATLAB R2019b (MathWorks, Natick, MA, USA). Spatial coregistration of the different modalities and segmentation of anatomical images for gray matter (GM), white matter (WM), and CSF were conducted using SPM12 (www.fil.ion.ucl.ac.uk/spm) with standard parameter settings. Leakage‐corrected CBV values were obtained using a reference curve approach and numerical integration [24–26]. Relative CBV (rCBV) values were calculated by assuming healthy WM values of 2.5% [27].

Post-processing of APTw images followed the vendor’s standard implementation.

### Image analysis

All images and parameter maps ([^18^F]-FET-PET, CBV, APTw) from a single patient were spatially normalized into the SRI24 atlas space [28] and resampled to 1 mm isotropic resolution using a rigid, mutual information-driven registration with the open-source ANTs software (https://stnava.github.io/ANTs/) [29]. Tumors were automatically segmented into necrosis, contrast-enhancing tumor, and FLAIR-hyperintense tumor, using the freely available BraTS Toolkit developed by us [30]. In brief, BraTS Toolkit ensembles several brain tumor segmentation algorithms, relying on a multimodal input of T1w, T1w with contrast, T2, and FLAIR images, and fuses the resulting candidate segmentations into a final consensus segmentation using SIMPLE fusion [31]. All registrations and segmentations were checked and — where necessary — corrected manually by KP (board-certified radiologist with 8 years of experience).

To limit subsequent image analysis to tumor regions with progressive signal alterations, we subtracted the tumor segmentation of the previous exam from the current segmentation and excluded necrotic areas.

From these segmentation areas, and using the coregistered sequences, we extracted pre-defined summary statistics (5th, 25th, 50th, 75th, and 95th percentile intensity, inter-quartile range, and Shannon entropy) as well as volumes of hot-spot areas delineated by the different modalities using a Python script. For hot-spot definition, we relied on pre-defined thresholds from the literature by using the following cut offs: APTw > 1.79 [12], tumor-background ratio > 2 for [^18^F]-FET-PET [32], and rCBV > 5.6 [33]. For estimation of the tumor-background ratio for the [^18^F]-FET-PET data, we relied on the white matter maps generated during CBV processing, excluding tumor areas, to calculate the background signal.

This feature vector was used as input for a Random Forest classifier [34], an ensemble-based machine learning algorithm which is known for its relative resistance to over-fitting as well as its ability to deal with correlated data. We used the scikit-learn implementation, leaving all parameters at their standard recommended default values, with a threefold cross-validation for obtaining unbiased estimates of the classifier performance. We also collected impurity-based feature importance to judge the contribution of the imaging modalities to the classifier’s performance.

### Statistical analysis

A receiver operator curve analysis was performed for the classifier based on all imaging data. Results are from a threefold cross-validation. Based on a cut off value of 0.5, we plotted confusion matrices and calculated sensitivity, specificity, and accuracy. Differences between the binary accuracy and the no-information rate in our data set were tested using a binomial test, with the no-information rate as success probability. Prediction accuracy between the different models were compared using a McNemar test.

## Results

Sixty-six patients (seventy-four cases) met our inclusion criteria. They had a median age of 55 years (range 54.91 ± 12.2), 45% of them were female (Table 1). Median interval between [^18^F]-FET-PET and MRI was 18.2 days. The reference standard used in this study, i.e., the final diagnosis of true progressive disease (PD, *n* = 57) and TRC (*n* = 17), was based on histopathology in 43 cases (resection or biopsy, n = 39 PD and n = 4 TRC) and on interdisciplinary board consensus based on follow-up MRI imaging (including APTw and CBV) after 3 months in 31 cases (n = 18 PD, n = 13 TRC) and according to the RANO criteria. In MRI, progression was suspected in 64 of 74 cases, whereas in [^18^F]-FET-PET, real progression was suspected in only 49/74 cases. In contrast, TRC was correctly identified only in 7/17 cases in MRI, but in 14/17 in PET.

**Fig. 2:**
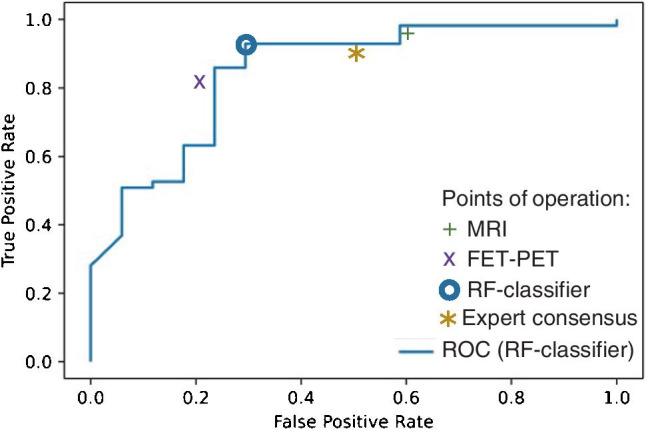
ROC curve of the Random Forest (RF) classifier, derived from the fully automated multimodal evaluation (AUC = 0.85) and a comparison of the point of operation with MRI, PET, and the expert consensus

ROC analysis for the identification of PD in our fully automated data analysis (results from a threefold cross-validation) yielded an area under the curve (AUC) of 0.85 (Fig. 2) with an accuracy of 0.86 (sensitivity 0.91, specificity 0.701). This model performance was significantly greater than the no-information rate in our data set (i.e., the rate of patients with PD; p = 0.03). Compared with these results, traditional assessment, relying either on MRI (accuracy: 0.82; sensitivity: 0.95; specificity: 0.41) or [^18^F]-FET-PET (accuracy: 0.81; sensitivity: 0.81; specificity: 0.82) as well as the interdisciplinary tumor board expert consensus (accuracy: 0.81; sensitivity: 0.81; specificity: 0.53), showed a lower diagnostic accuracy not significantly above chance (Table 2). Upon comparison of the machine learning model with predictions based on either MRI (p = 0.774) or [^18^F]-FET-PET (p = 0.424), we noted no significant differences.

**Table 2:** Confusion matrices for MRI (accuracy: 0.82 (95% CI 0.72–0.9), sensitivity: 0.95 (95% CI 0.85–0.99), specificity: 0.41 (95% CI 0.18–0.67)), [^18^F]-FET-PET (accuracy: 0.81 (95% CI 0.7–0.89), sensitivity: 0.81 (95% CI 0.68–0.9), specificity: 0.82 (95% CI 0.57–0.96)), the Random Forest classifier derived from fully automated multimodal evaluation (accuracy: 0.86 (95% CI 0.77–0.93), sensitivity: 0.91 (95% CI 0.81–0.97), specificity: 0.71 (95% CI 0.44–0.9)), and expert consensus in our institutional tumor board (accuracy: 0.81 (95% CI 0.7–0.89), sensitivity: 0.89 (95% CI 0.79–0.96), specificity: 0.53 (95% CI 0.28–0.77))

	Suspected PD	Suspected TRC		
MRI				
True PD	54	3	57	
True TRC	10	7	17	
	64	10	74	
PET				
True PD	46	11	57	
True TRC	3	14	17	
	49	25	74	
Random Forest classifier				
True PD	52	5		57
True TRC	5	12		17
	57	17		74
Expert consensus				
True PD	51	6		57
True TRC	8	9		17
	59	15		74

**Fig. 3:**
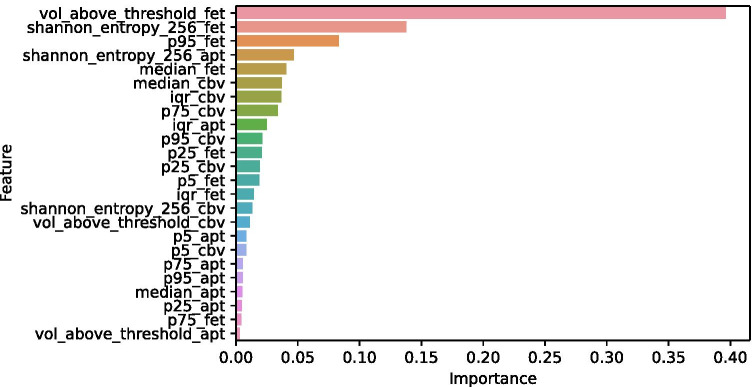
Feature importance. The length of the bar indicates relative importance (summing to 1) of each input feature for classifier performance, i.e., longer bars indicate relatively higher importance

**Fig. 4:**
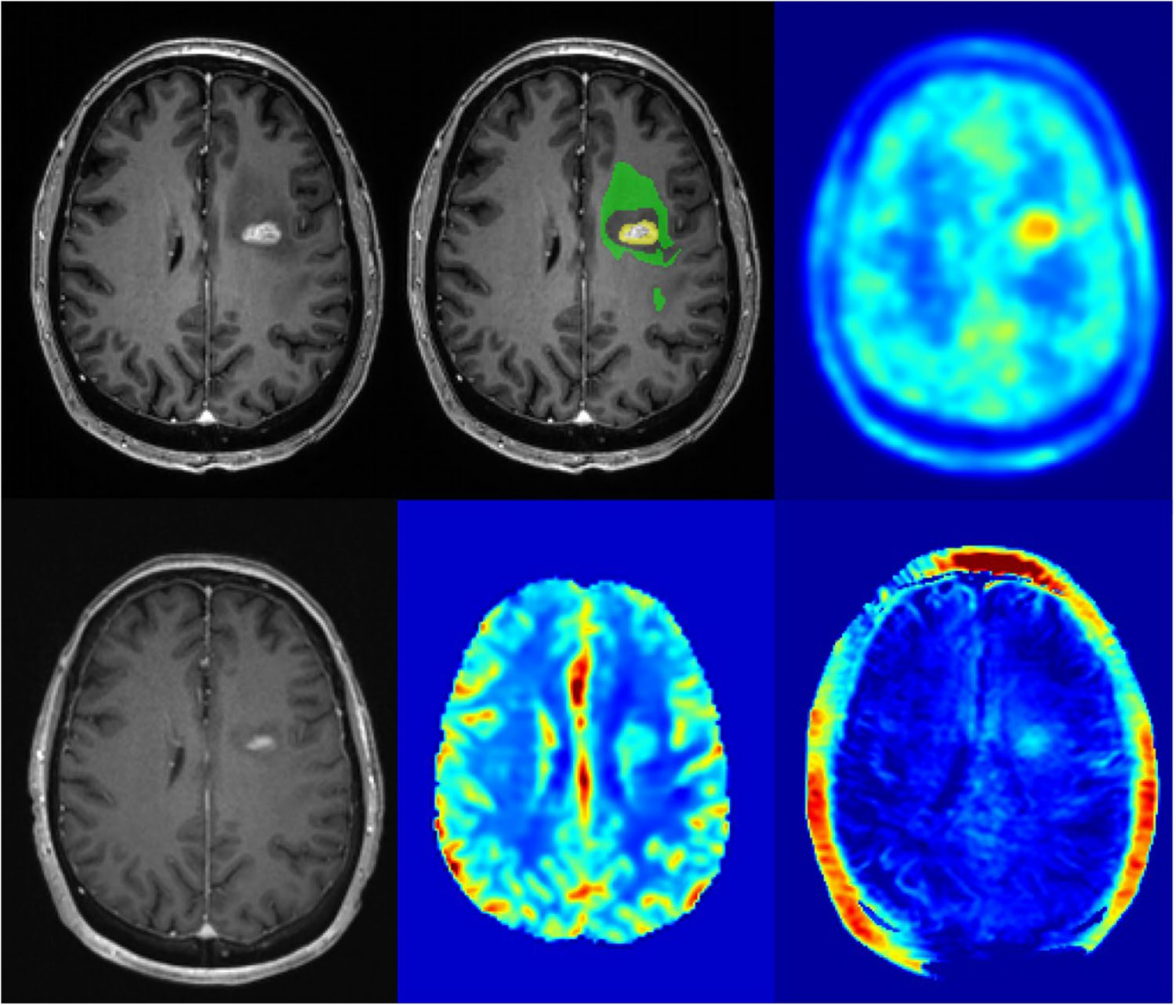
Example images of progressive disease in a 65-year-old female patient with left frontal GBM and a new contrast enhancement superior to the former resection area (upper left: ce T1-w, lower left: previous time point, upper middle: automated segmentation overlay (green: new FLAIR edema area; yellow: new ce area), lower middle: CBV, upper right: [^18^F]-FET-PET, lower right APTw)

**Fig. 5:**
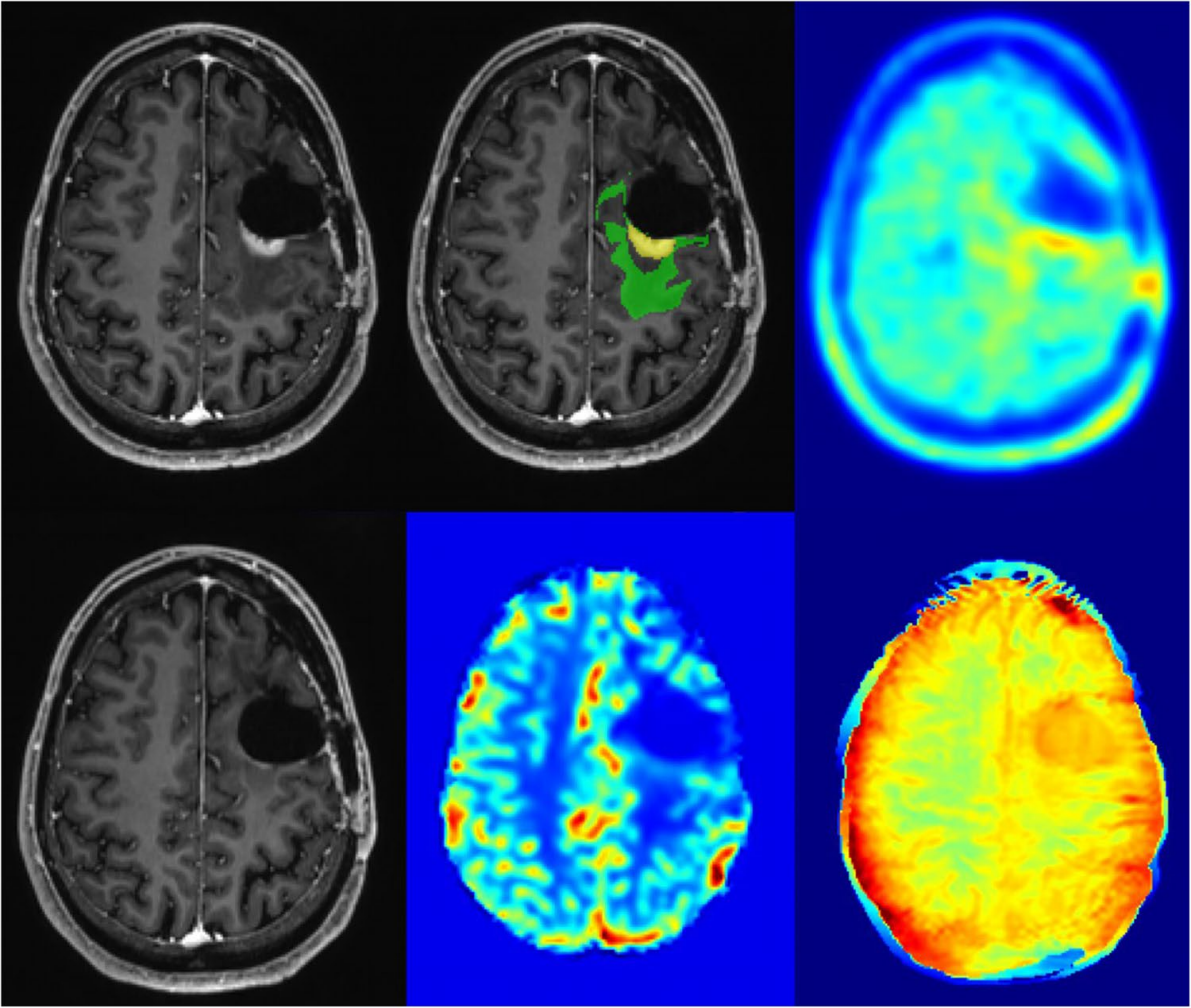
Example images of TRC in a 34-year-old male with a new contrast enhancing focus next to the resection cavity after therapy of a left frontal GBM (upper left: ce T1-w, lower left: previous time point, upper middle: automated segmentation overlay (green: new FLAIR edema area; yellow: new ce area), lower middle: CBV, upper right: [^18^F]-FET-PET, lower right APTw)

Upon inspection of the final model and the individual contribution of imaging features (Fig. 3), imaging information derived from [^18^F]-FET-PET data contributed most importantly to the classifier. However, also features from APTw and CBV maps ranked high, indicating a reliance of the classifier on joint imaging information (Figs. 4 and 5).

## Discussion

To allow for objective, user-independent assessment of therapy response, we have developed an automated, joint image analysis of [^18^F]-FET-PET and advanced MR imaging techniques. The classification accuracy of the Random Forest classifier was 0.86 (sensitivity 0.91, specificity 0.71) and therefore significantly above the no-information rate of 0.77 (p = 0.03) compared to an accuracy of 0.82 for MRI, 0.81 for [^18^F]-FET-PET, and 0.81 for expert consensus. The single-most important variable was [^18^F]-FET-PET hot-spot volume, with relevant contribution from all imaging modalities.

Diagnosis and in particular response assessment of brain tumors is strongly based on imaging, especially MRI techniques [35–37], because histological confirmation often cannot be realized easily and bears substantial risks. In the future, artificial intelligence (AI) has the potential to improve image-based diagnosis [38] and augment clinical decision-making in the management of oncologic patients [39]. However, assessing the rich, multimodal imaging information is a complex task, and in particular when MR and [^18^F]-FET-PET [40, 41] images need to be assessed jointly, requires expertise from multiple disciplines. In many institutions, interdisciplinary analysis of these images (and the patient history) in tumor boards has therefore become standard of care. Even there however, the extraction of (semi-)quantitative information from these images (such as tumor-background ratio from [^18^F]-FET-PET images [42]) is limited by its inherent inter-observer variability and the tediousness of manually delineating (progressive) tumor areas on images. Promising to overcome these challenges, advances in automated image analysis offer the perspective of objective, multimodal assessment of tumor response [43, 44]. As highlighted in the Brain Tumor Segmentation Challenge (BraTS) [45], machine learning-based glioma segmentation has now reached a point where the algorithms’ performance is non-inferior to human raters. This has been shown to facilitate objective response assessment according to the RANO criteria in a large, prospective trial cohort [38]. In parallel, machine learning algorithms excel at the (non-linear) analysis of multimodal data and thus offer a means to harvest the wealth of information contained in medical imaging data [20, 43].

Assessing the synergistic value of multiple imaging techniques for glioma diagnosis and response assessment has been investigated by several groups. Park et al. [46] for example concluded that adding APT imaging to conventional and perfusion MRI significantly improves the diagnostic performance for differentiating true progression from pseudoprogression. Furthermore, they found that a combination of contrast-enhanced T1w, nCBV90, and APT90 resulted in greater diagnostic accuracy for differentiating true progression from pseudoprogression than the combination of contrast-enhanced T1w and nCBV90 alone. Liesche et al. [47] showed that ^18^F-fluoroethyl-tyrosine uptake is correlated with amino acid transport and neovascularization in treatment-naive glioblastomas. Following these results, a study by Schön et al. [48] compared the synergism of amino acid PET, amide proton transfer, and perfusion-weighted MRI in newly diagnosed gliomas. They found that the overlap between APTw/CBV is relevantly lower than between APTw/FET, indicating a potential synergistic value of combining APTw and CBV information. This was further corroborated by investigating the stereotactic biopsies, where they found a more pronounced association of CBV and vascularity (compared with APTw) on the one side, and a stronger correlation of APTw with cellularity on the other side. Their findings also underlined the future diagnostic potential of a multimodal imaging concept in making oncogenic processes visible and supporting decision-making in clinically challenging situations such as grading gliomas or differentiating radiation necrosis from real progression. Along this line, such information-rich data sets serve as an ideal basis for training machine learning classifiers which are able to (non-linearly) integrate the multimodal input data, as we and others have previously demonstrated [49]. Inspecting the final model and individual contribution of imaging features in our study, imaging information derived from [^18^F]-FET-PET data contributed most importantly to the classifier (Fig. 3). This predominance of [^18^F]-FET-PET information has been suggested by others [50]. However, also features from APTw and CBV maps ranked high, indicating a reliance of the classifier on joint imaging information. The importance of integrating multimodal information is also highlighted by the results for our algorithm compared with the individual assessment of ^18^F]-FET-PET and MRI (Fig. 2, Table 2): While in MRI, there was a bias towards diagnosing PD (10 cases of TRC mis-diagnosed as PD), whereas in [^18^F]-FET-PET, the opposite was the case (11 cases of PD mis-diagnosed as TRC). This was balanced out in the multimodal Random Forest classifier (5 vs. 5) and — to a lesser degree — also in the expert consensus including both departments of neuroradiology and nuclear medicine (8 vs. 6).

Up to now, only few studies are available that involve machine learning techniques to differentiate between treatment-related changes and real tumor progression. An SVM (support vector machine) classifier has been trained to diagnose pseudoprogression vs. recurrence in patients with glioma treated with surgery and chemotherapy. In their study containing 31 patients, Hu et al. showed a sensitivity and specificity of the classifier for pseudoprogression of 89.91 and 93.72%, respectively, with AUC of 0.94; with DWI and rCBV as the best predictor image sequences [51]. A CNN has been developed to differentiate true progressive disease from pseudoprogression in patients with a GBM status post chemo-radiation and surgery with a performance of AUC = 0.83, comparable to our MR-only results [3]. However, these studies still required manual labeling of tumor regions (or in the case of the study by Jang et al. the selection of tumor-bearing slices) for subsequent analysis. In contrast, our method is fully automated, from longitudinal tumor segmentation and feature extraction to classification.

In our study, we decided not to limit our analysis to patients with suspected pseudoprogression (i.e., those with new or progressive contrast enhancement following radiotherapy) but rather include all patients where response assessment proved difficult enough to warrant [^18^F]-FET-PET imaging and discussion in our interdisciplinary tumor board. Despite these broad inclusion criteria, 23% of patients showed TRC; a number in line with the literature [1]. Further, we have included eight patients twice. While this might induce a bias in our model, this choice reflects real clinical practice potentially better than opposed to strict inclusion criteria, which tend to limit generalizability of results and possibly bias models towards patients meeting these strict inclusion criteria. Importantly though, these eight patients had a change of treatment between both time points included in our study and therefore were not included twice for the same therapy.

As our results are promising, the present study has some limitations. First of all, we used a unicentric study as well as a relatively small sample size. In particular, our results were only validated on internal data using a cross-validation approach. The lack of an (ideally external) test set as therefore to be considered when interpreting our results. How readily our models generalize to data from other hospitals/scanners if therefore also not clear. We opted for an advanced imaging protocol — including APTw imaging — which might limit the immediate broad applicability of our findings. On the other hand, our approach thus highlights the value of multimodal imaging, capturing different aspects of tumor biology, and image analysis. When excluding APTw imaging, we observe a model AUC of 0.82. Future studies will be necessary to determine the best combination of imaging modalities; however, upon inspection of feature importance, we also find relevant information from APTw. We included FET-PET data from both a PET CT scanner as well as a PET MR scanner. While this improves the variability in our data and potentially also generalizability, we did not assess the influence of the differences in attenuation correction. For modeling our data, we chose a Random Forest, which is a well-established machine learning model for classification in the presence of (potentially) correlated input data. While modern deep learning approaches are able to learn even more complex decision boundaries and might potentially outperform a Random Forest, we were lacking enough data to train (and in particular independently test) such a model. Also, in particular, the segmentation ensemble necessitates the presence of additional hardware (GPU). Integration of such algorithms into clinical routine is a further challenge to be solved. While histology is considered gold standard for response assessment, tumor progression and TRC were determined by follow-up imaging in some cases and could not be validated by histological data. Finally, as most of the patients diagnosed with PD previously underwent radiotherapy, histopathologic samples of real progression in reality often show a mixture of areas of true progression as well as (microscopic) therapy-related changes, a fact neither MRI nor [^18^F]-FET-PET is able to resolve up to now. As tumor heterogeneity is a challenging fact, further studies — especially with the help of automated response assessments as a promising tool — have to be conducted to improve the detection and resolution of local tumor heterogeneity.

## Conclusions

Predicting tumor biology and response on imaging using AI is promising to play an important role in future practice. Our study shows that [^18^F]-FET-PET, multiparametric MRI with APTw and DSC perfusion parameters can be combined in a fully automated analysis to help objectively evaluate treatment response in gliomas and may therefore aid in the optimal care of these patients. The promise and performance of AI techniques in daily clinical practice and their effect on patient outcomes warrant further development.

## Supplementary Information

Below is the link to the electronic supplementary material.

Supplementary file1 (PDF 421 KB)

## Data Availability

The data sets generated and analyzed during the current study are available from the corresponding author on reasonable request.

## Code availability

N/A
